# Supplementary material for: Genome-wide DNA methylation and gene expression in human placentas derived from assisted reproductive technology
Source: Commun Med (Lond). 2024 Dec 19;4:267. doi: 10.1038/s43856-024-00694-6 (PMC11659305; doi:10.1038/s43856-024-00694-6)
Supplement: Supplementary file 2 — Supplementary Information [file 43856_2024_694_MOESM2_ESM.pdf]

# Supplementary Information:

## Supplementary Information for Genome-wide DNA methylation and gene expression in human placentas derived from Assisted Reproductive Technology

Pauliina Auvinen[1], Jussi Vehviläinen[1], Karita Rämö[1], Ida Laukkanen[1], Heidi Marjonen-Lindblad[1], Essi Wallén[1], Viveca Söderström-Anttila[2], Hanna Kahila[3], Christel Hydén-Granskog[3], Timo Tuuri[3], Aila Tiitinen[3], Nina Kaminen-Ahola[1]

*1. Environmental Epigenetics Laboratory, Department of Medical and Clinical Genetics, Medicum, University of Helsinki, Helsinki, Finland*

*2. The Family Federation of Finland, Fertility Clinic and University of Helsinki, Helsinki, Finland*

*3. Department of Obstetrics and Gynecology, Helsinki University Hospital and University of Helsinki, Helsinki, Finland*

**Supplementary Fig. 1:** Parental infertility diagnoses of ART, IUI, and SF newborns.

**Supplementary Fig. 2:** DNAm profiles of control, ART, and SF placentas at *DLK1-DIO3* ICR.

**Supplementary Fig. 3:** Cell type composition in placental samples.

**Supplementary Fig. 4:** Comparison of cell type compositions between control, ART, IUI, and SF placental samples.

**Supplementary Fig. 5:** GWAM comparison between all control, ART, IUI, and SF placentas.

**Supplementary Fig. 6:** Comparison of RE DNAm between control, ART, IUI, and SF placentas.

**Supplementary Fig. 7:** GWAM comparison between male control and ART placentas.

**Supplementary Fig. 8:** GWAM comparison between female control and ART placentas.

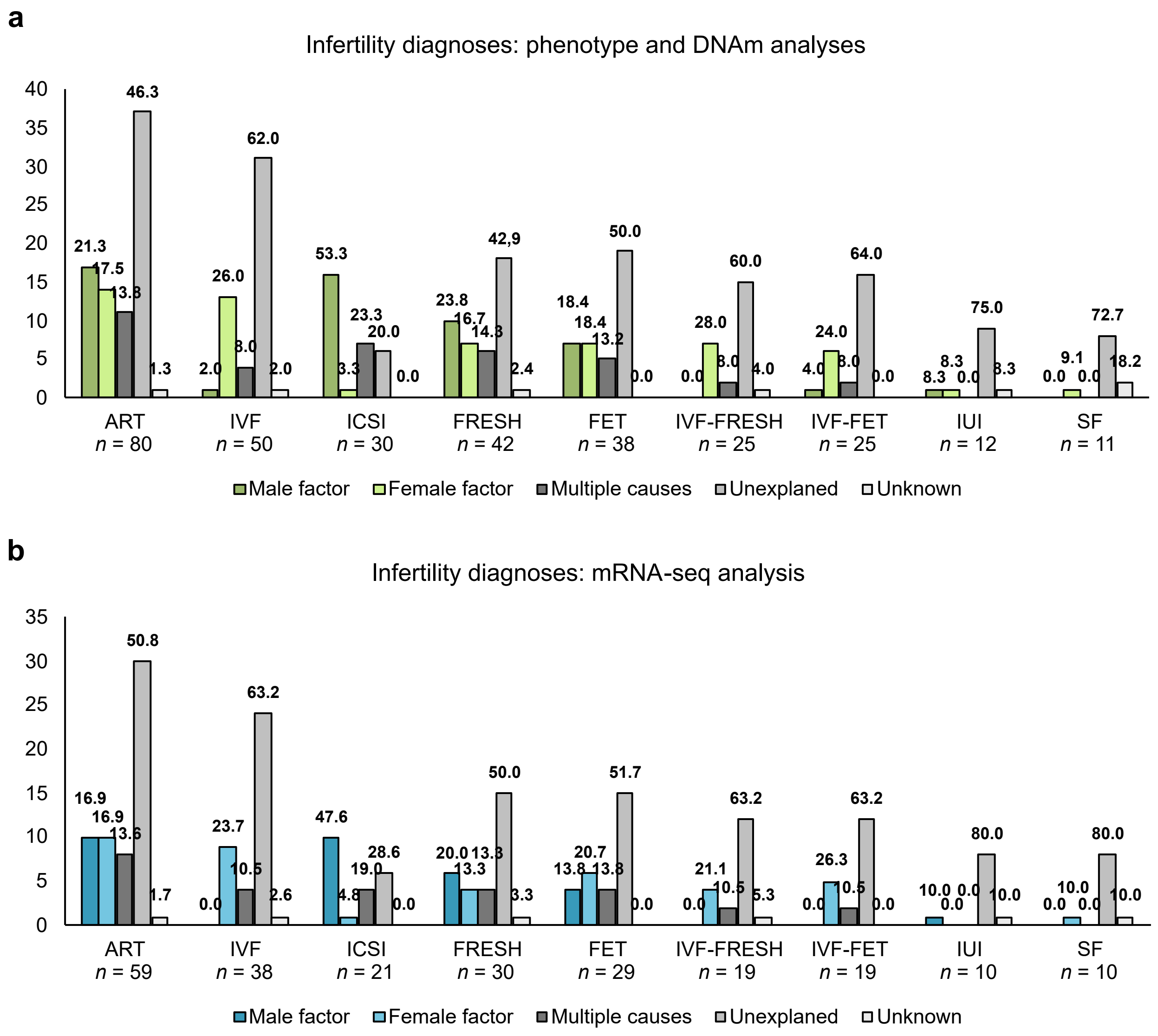


**Supplementary figure 1: Parental infertility diagnoses of ART, IUI, and SF newborns.** The proportions of categorized infertility diagnoses within study groups in **a** phenotype and genome-wide DNAm analysis, and in **b** genome-wide mRNA-seq analysis. The percentages of categorized diagnoses in each group are indicated above the bars. The proportions of the diagnoses in sex-specific analyses are not presented. In cases where the diagnosis was "unknown", the diagnostic information was missing.

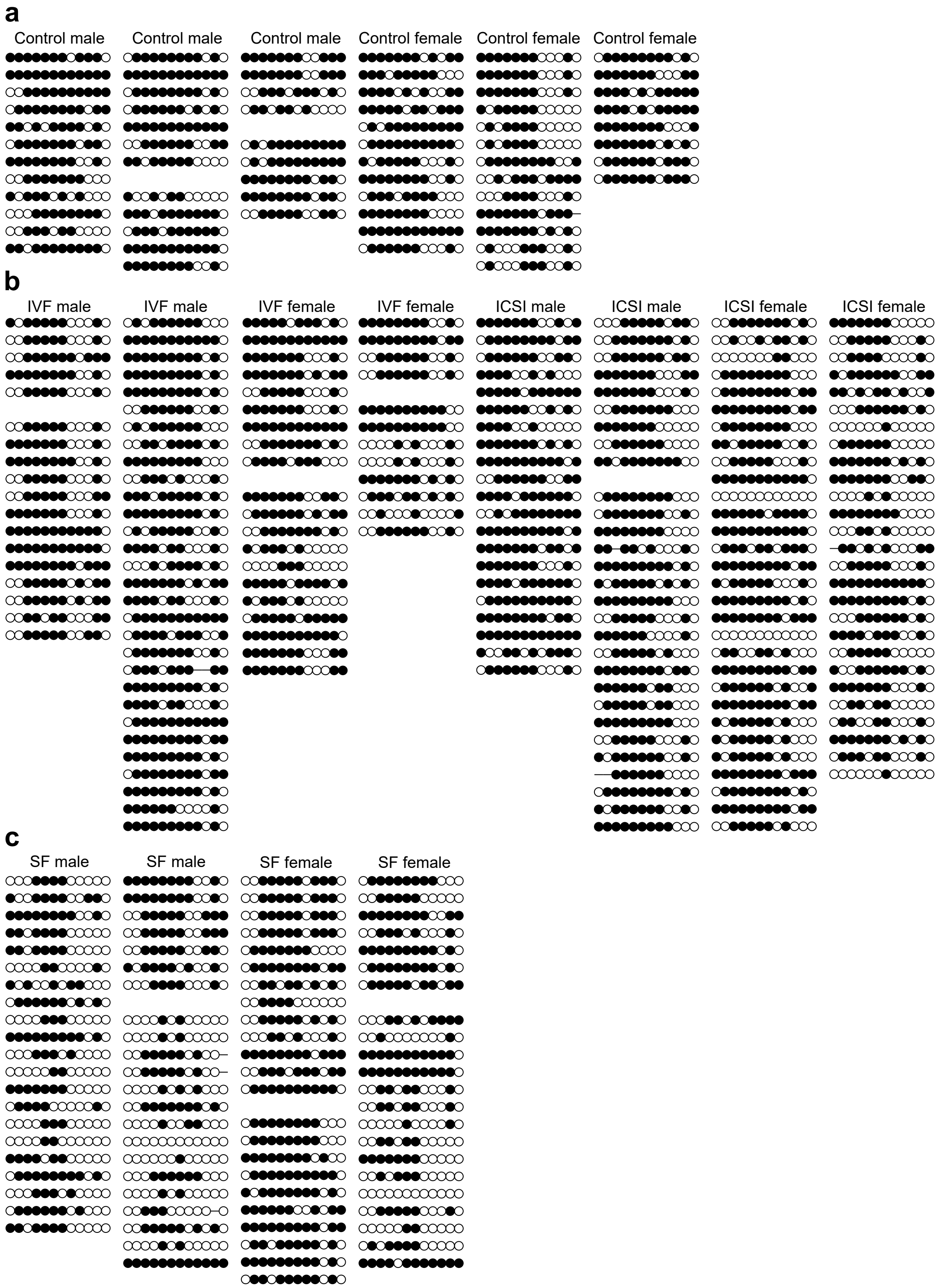


**Supplementary figure 2: DNAm profiles of control, ART, and SF placentas at *DLK1-DIO3* ICR.** Visualization of DNAm profiles at 12 CpG sites (chr14:101,277,375-101,277,826) in *DLK1-DIO3* ICR in **a** control ($n$ = 6), **b** IVF ($n$ = 4) and ICSI ($n$ = 4), as well as **c** in SF ($n$ = 4) male and female placentas. Paternal and maternal alleles are distinguished according to rs1884539(A/G) and rs75998174(A/G) polymorphisms when possible. Methylated and unmethylated CpGs are shown in black and white circles, respectively.

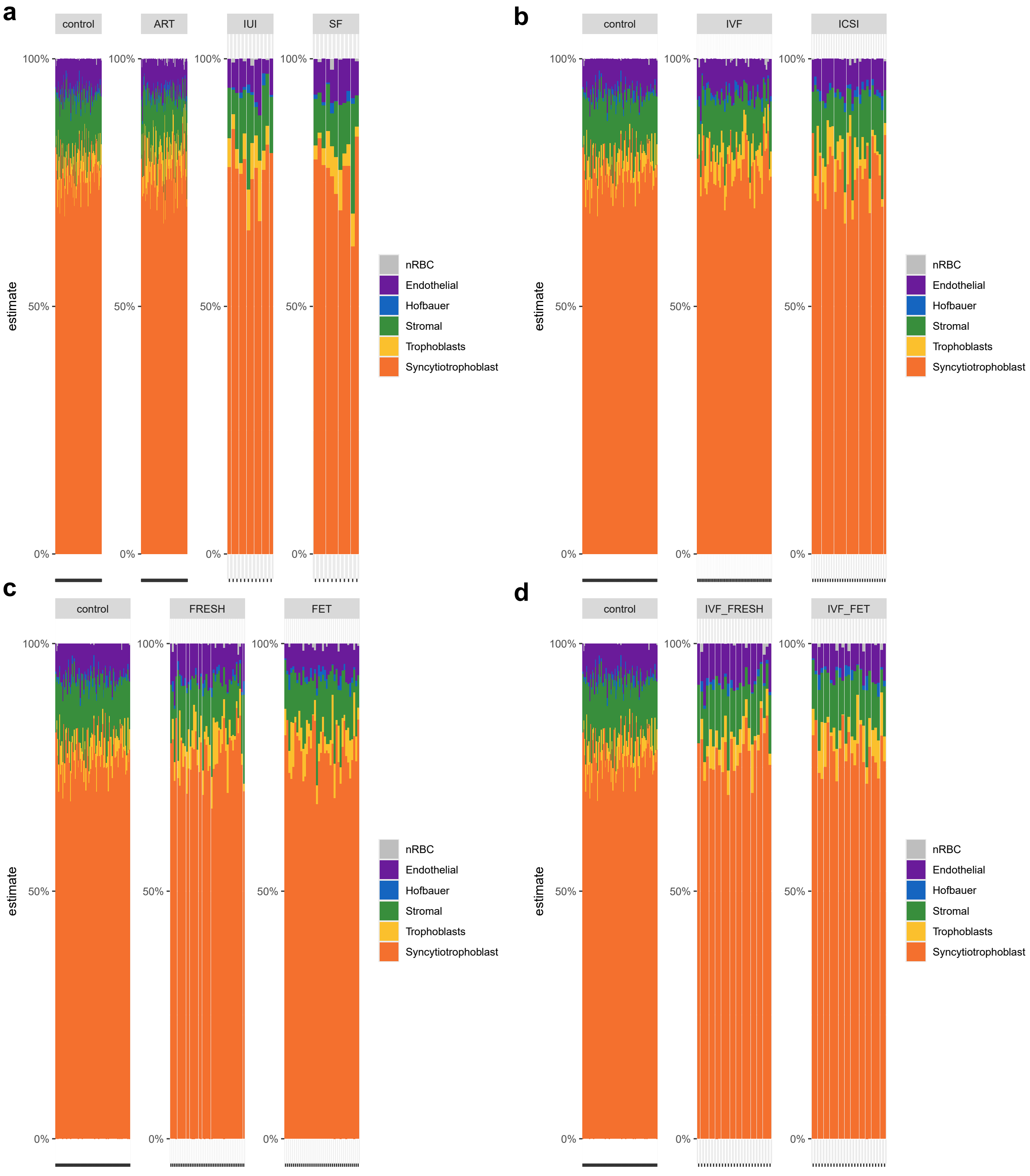


**Supplementary figure 3: Cell type composition in placental samples.** Cell type composition in **a** control, ART, IUI, and SF **b** control, IVF, and ICSI, **c** control, FRESH, and FET as well as in **d** control, IVF-FRESH, and IVF-FET placental samples. In addition to the five major placental cell types, the fragments of nucleated red blood cells (nRBCs) are shown. Control *n* = 77, ART *n* = 80, IVF *n* = 50, ICSI *n* = 30, FRESH *n* = 42, FET *n* = 38, IVF-FRESH *n* = 25, IVF-FET *n* = 25, IUI *n* = 12, and SF *n* = 11.

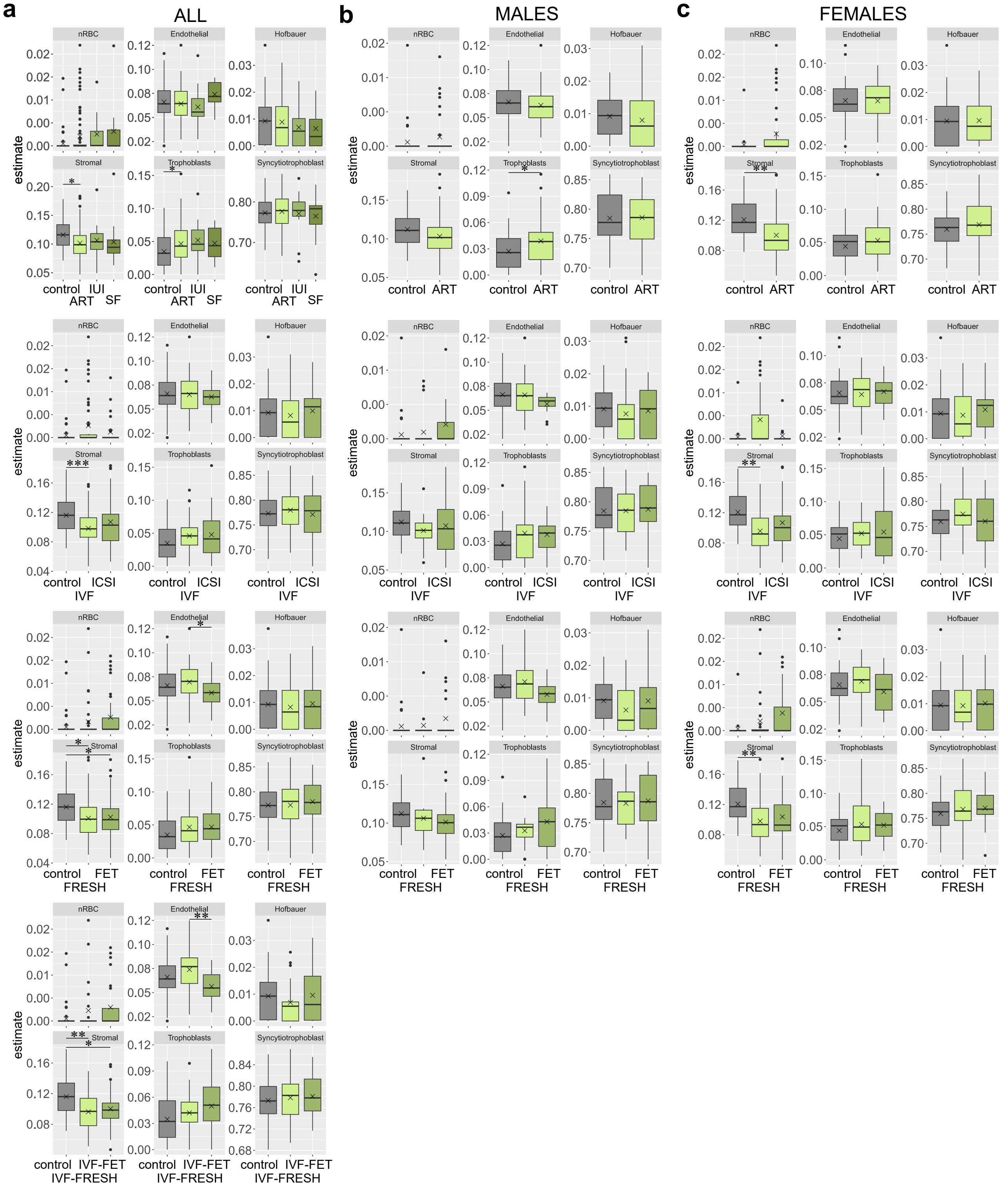


**Supplementary figure 4: Comparison of cell type compositions between control, ART, IUI, and SF placental samples.** Comparison of cell type compositions between **a** all, **b** male, and **c** female control, ART, ART subgroup, IUI, and SF placental samples presented by box plots. Statistical differences of nucleated red blood cell (nRBC) fragments between the study groups were not calculated. *$P$ < 0.05 two-sided $t$-test or One-Way ANOVA followed by Tukey's HSD test, **$P$ < 0.01, two-sided $t$-test or One-Way ANOVA followed by Tukey's HSD test, and ***$P$ < 0.001, Kruskal-Wallis test followed by Wilcoxon Rank Sum Exact test. Control $n$ = 77 (42 males/35 females), ART $n$ = 80 (36/44), IVF $n$ = 50 (24/26), ICSI $n$ = 30 (12/18), FRESH $n$ = 42 (14/28), FET $n$ = 38 (22/16), IVF-FRESH $n$ = 25, IVF-FET $n$ = 25, IUI $n$ = 12, and SF $n$ = 11.

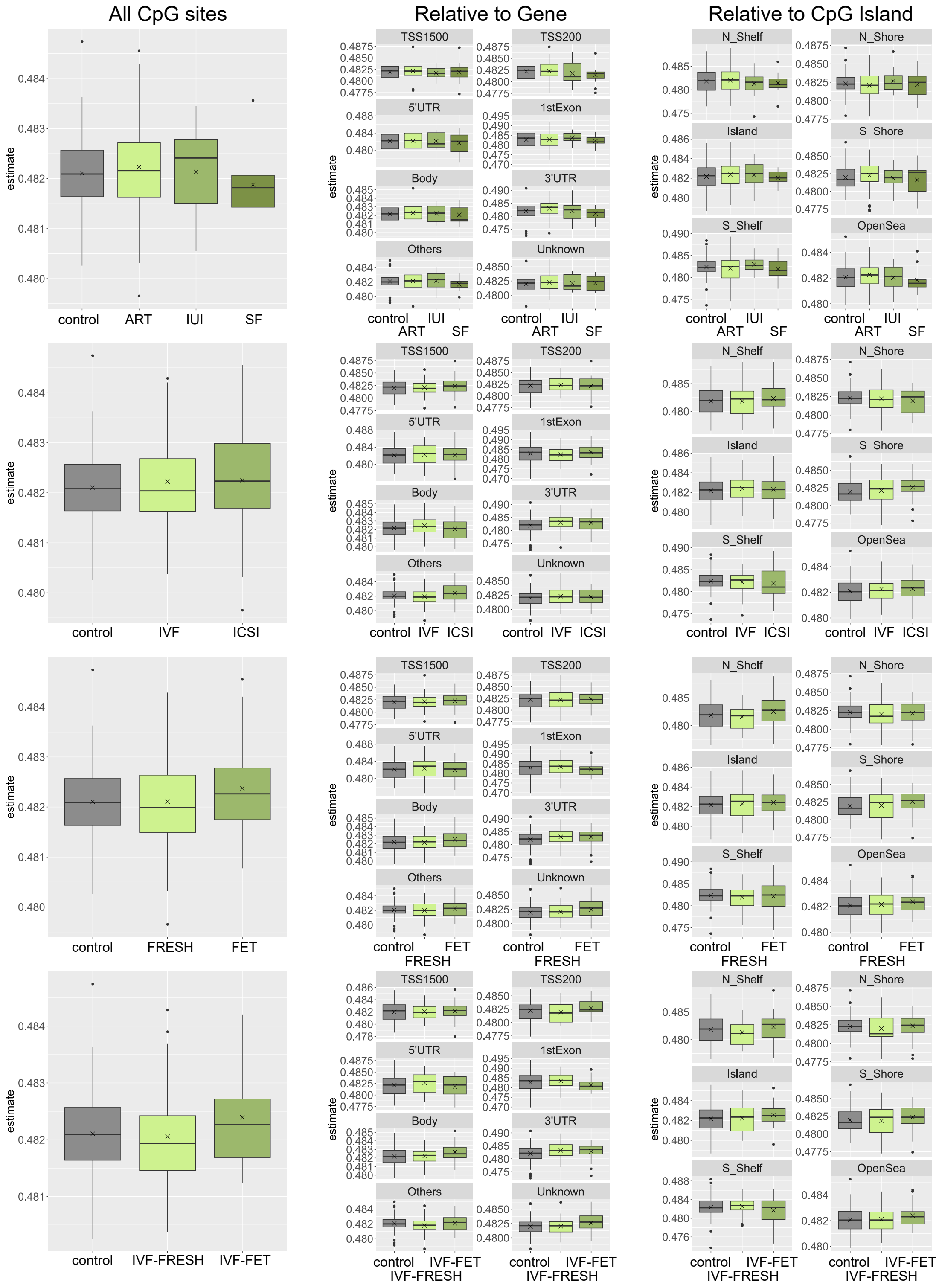


**Supplementary figure 5: GWAM comparison between all control, ART, IUI, and SF placentas.** Comparison of DNAm in all probes, in relation to gene, and in relation to CpG island between all control, ART, ART subgroup, IUI, and SF placentas presented by box plots. Control *n* = 77, ART *n* = 80, IVF *n* = 50, ICSI *n* = 30, FRESH *n* = 42, FET *n* = 38, IVF-FRESH *n* = 25, IVF-FET *n* = 25, IUI *n* = 12, and SF *n* = 11. TSS1500: 1500 bp upstream of transcription start site, TSS200: 200 bp upstream of TSS, UTR: untranslated region, N_shelf: north shelf, N_shore: north shore, S_shore: south shore, S_shelf: south shelf.

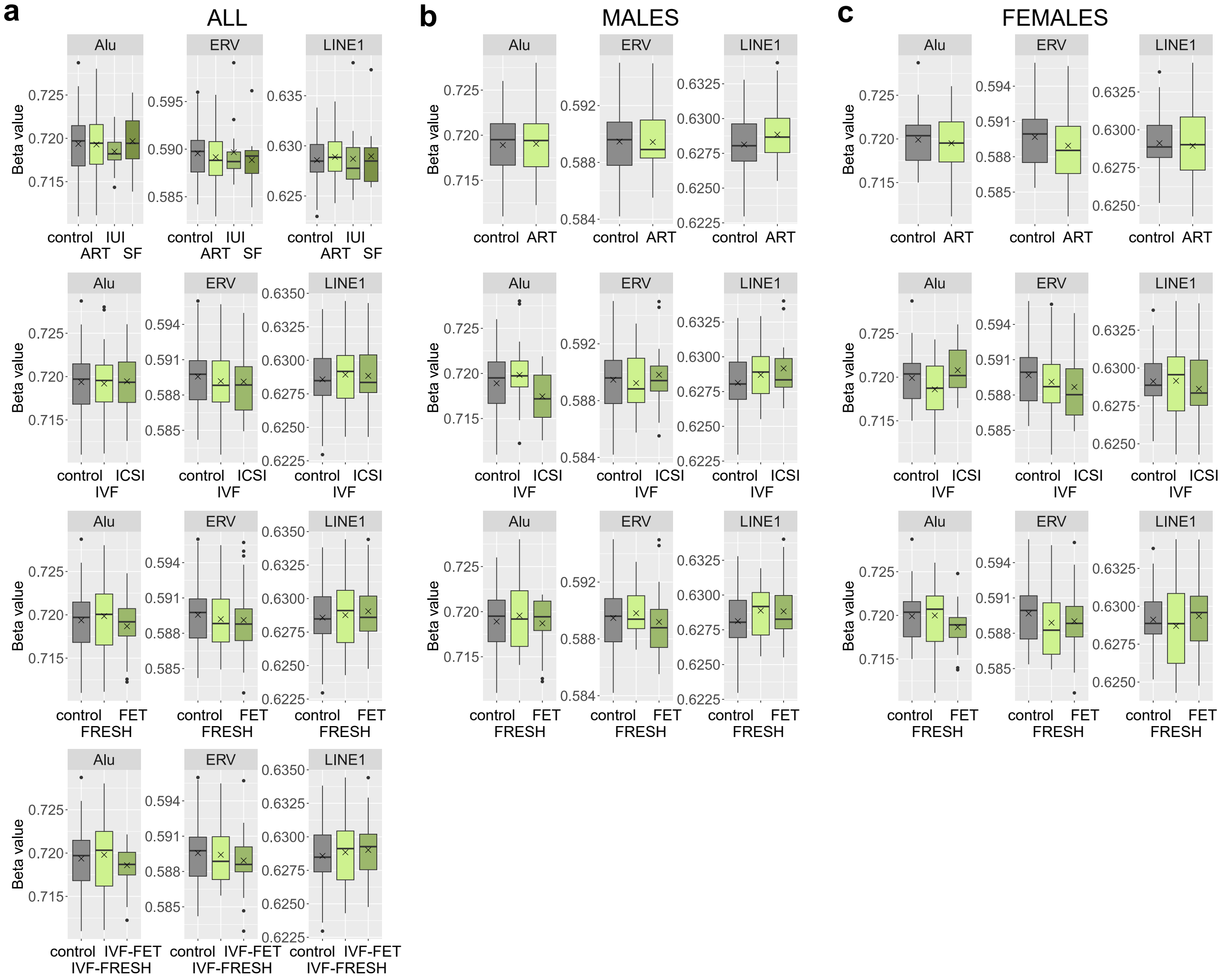


**Supplementary figure 6: Comparison of RE DNAm between control, ART, IUI, and SF placentas.** Comparison of DNAm in Alu, LINE1, and LTR repetitive regions between **a** all, **b** male, and **c** female control, ART, ART subgroup, IUI, and SF placentas presented by box plots. Control *n* = 77 (42 males/35 females), ART *n* = 80 (36/44), IVF *n* = 50 (24/26), ICSI *n* = 30 (12/18), FRESH *n* = 42 (14/28), FET *n* = 38 (22/16), IVF-FRESH *n* = 25, IVF-FET *n* = 25, IUI *n* = 12, and SF *n* = 11.

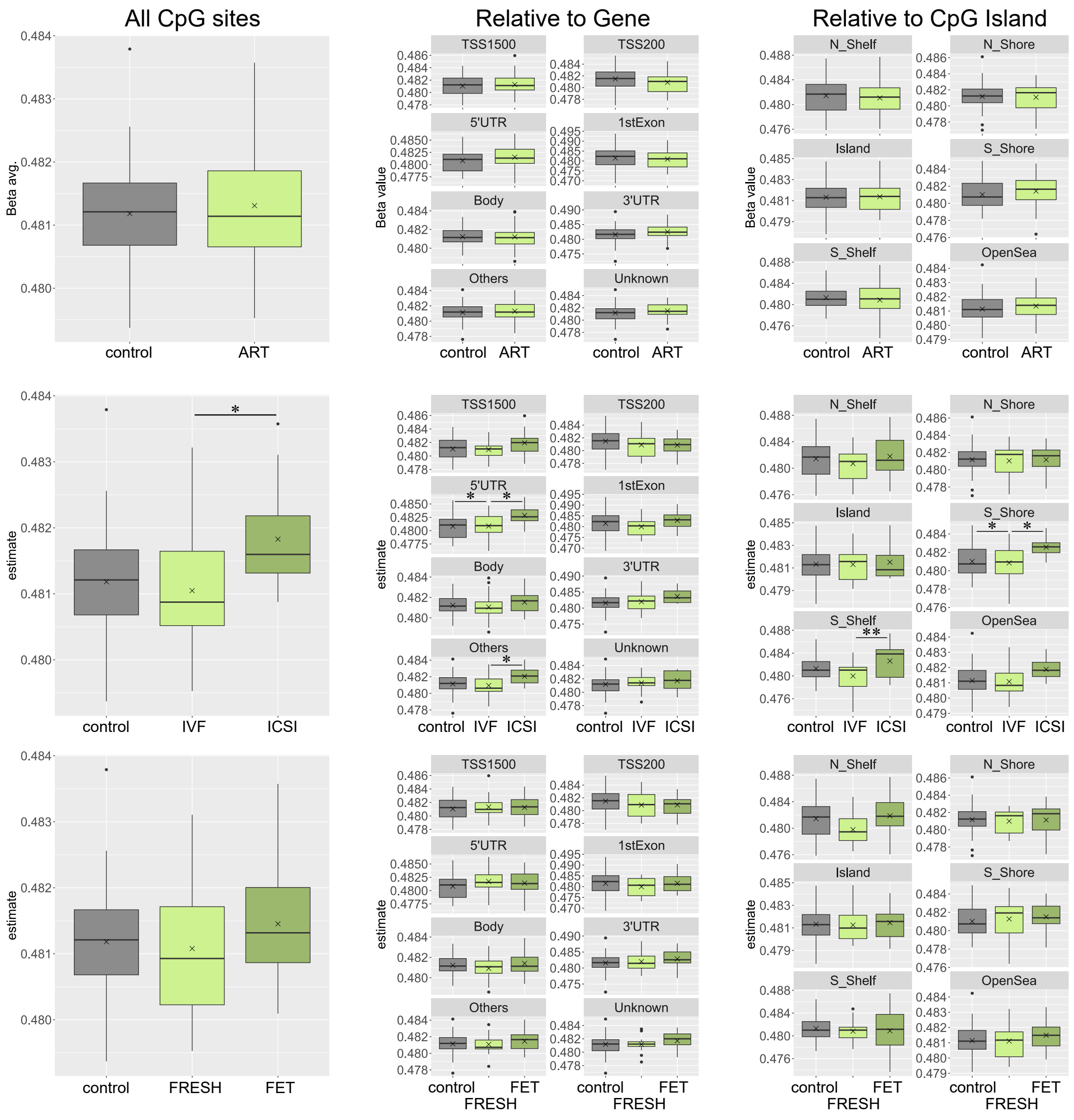


**Supplementary figure 7: GWAM comparison between male control and ART placentas.** Comparison of DNAm in all probes, in relation to gene, and in relation to CpG island between male control, ART, and ART subgroup placentas presented by box plots. **P* < 0.05 and ***P* < 0.01, One-Way ANOVA followed by Tukey's HSD test. Control *n* = 42, ART *n* = 36, IVF *n* = 24, ICSI *n* = 12, FRESH *n* = 14, and FET *n* = 22. TSS1500: 1500 bp upstream of transcription start site, TSS200: 200 bp upstream of TSS, UTR: untranslated region, N_shelf: north shelf, N_shore: north shore, S_shore: south shore, S_shelf: south shelf.

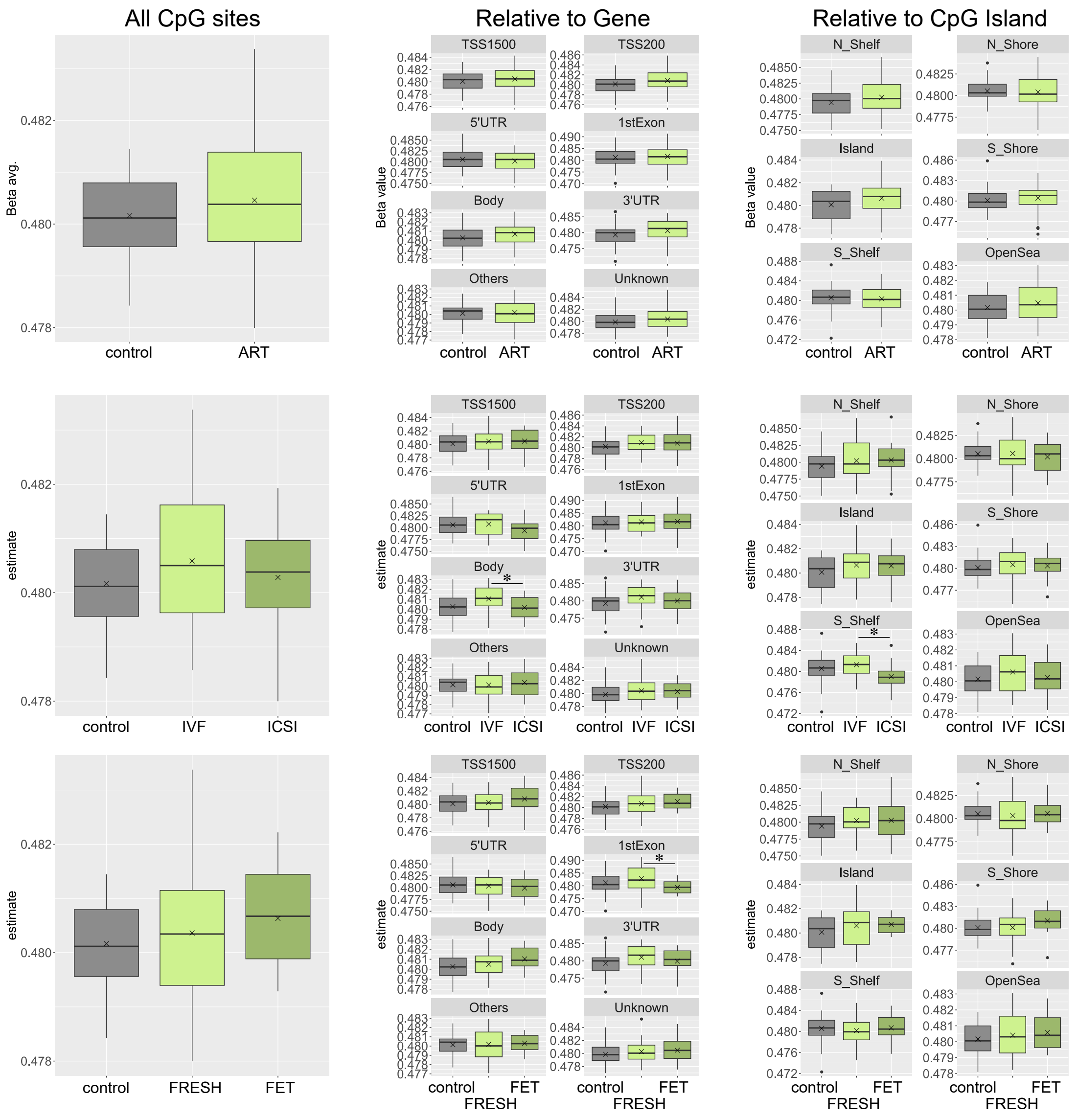


**Supplementary figure 8: GWAM comparison between female control and ART placentas.** Comparison of DNAm in all probes, in relation to gene, and in relation to CpG island between female control, ART, and ART subgroup placentas presented by box plots. **P* < 0.05, One-Way ANOVA followed by Tukey's HSD test. Control *n* = 35, ART *n* = 44, IVF *n* = 26, ICSI *n* = 18, FRESH *n* = 28, FET *n* = 16. TSS1500: 1500 bp upstream of transcription start site, TSS200: 200 bp upstream of TSS, UTR: untranslated region, N_shelf: north shelf, N_shore: north shore, S_shore: south shore, S_shelf: south shelf.
