# Supplementary material for: Genome-wide DNA methylation and gene expression in human placentas derived from assisted reproductive technology
Source: Commun Med (Lond). 2024 Dec 19;4:267. doi: 10.1038/s43856-024-00694-6 (PMC11659305; doi:10.1038/s43856-024-00694-6)
Supplement: Supplementary file 3 — Description of Additional Supplementary Files [file 43856_2024_694_MOESM3_ESM.pdf]

Description of Additional Supplementary Files

Supplementary Data: Genome-wide DNA methylation and gene expression in human placentas derived from Assisted Reproductive Technology

Supplementary Data 1: General characteristics of control, ART, IUI, and SF newborns as well as their mothers included in the phenotype and genome-wide DNAm analysis

Supplementary Data 2: Parental infertility diagnoses of ART, IUI and SF newborns

Supplementary Data 3: General characteristics of control, ART, IUI, and SF newborns as well as their mothers included in the genome-wide mRNA-seq analysis

Supplementary Data 4: ART-associated differentially methylated CpGs

Supplementary Data 5: ART-associated DMRs

Supplementary Data 6: GO and KEGG enrichment analyses for ART-associated DMRs

Supplementary Data 7: ART-associated DEGs

Supplementary Data 8: Normalized mRNA-seq counts of DLK1, TRIM28, and NOTCH3

Supplementary Data 9: Gene expression correlations in control, ART, IUI and SF placentas

Supplementary Data 10: GO enrichment analysis for ART-associated DEGs

Supplementary Data 11: IUI- and in vitro culture-associated differentially methylated CpGs and DMRs

Supplementary Data 12: IUI-, SF-, and in vitro culture-associated DEGs

Supplementary Data 13: IVF-associated differentially methylated CpGs

Supplementary Data 14: IVF-associated DMRs

Supplementary Data 15: GO and KEGG enrichment analyses for IVF-associated DMRs

Supplementary Data 16: ICSI-associated differentially methylated CpGs

Supplementary Data 17: Common ART-associated alterations between IVF and ICSI placentas

Supplementary Data 18: IVF- and ICSI-associated DEGs

Supplementary Data 19: GO enrichment analysis for ICSI-associated DEGs

Supplementary Data 20: FRESH-associated differentially methylated CpGs

Supplementary Data 21: FET-associated differentially methylated CpGs

Supplementary Data 22: Common ART-associated alterations between FRESH and FET placentas

Supplementary Data 23: FRESH-associated DMRs

Supplementary Data 24: FET-associated DMRs

Supplementary Data 25: GO and KEGG enrichment analyses for FRESH-associated DMRs

Supplementary Data 26: GO and KEGG enrichment analyses for FET-associated DMRs

Supplementary Data 27: L FRESH- and FET-associated DEGs

Supplementary Data 28: IVF-FRESH- and IVF-FET-associated differentially methylated CpGs

Supplementary Data 29: IVF-FRESH- and IVF-FET-associated DMRs

Supplementary Data 30: ART-associated sex-specific DNAm alterations

Supplementary Data 31: ART-associated sex-specific gene expression alterations

Supplementary Data 32: Correlations between placental gene expression, newborn phenotype, and maternal characteristics

Supplementary Data 33: ART-associated placental imprinting

Supplementary Data 34: DNAm levels at DLK1-DIO3 ICR in control, ART, IVF, ICSI, and SF placentas
