# Supplementary material for: Genome-wide DNA methylation and gene expression in human placentas derived from assisted reproductive technology
Source: Commun Med (Lond). 2024 Dec 19;4:267. doi: 10.1038/s43856-024-00694-6 (PMC11659305; doi:10.1038/s43856-024-00694-6)
Supplement: Supplementary file 5 — Reporting Summary [file 43856_2024_694_MOESM5_ESM.pdf]

nature portfolio

Corresponding author(s): Nina Kaminen-Ahola

Last updated by author(s): Sep 11, 2024

Reporting Summary

Nature Portfolio wishes to improve the reproducibility of the work that we publish. This form provides structure for consistency and transparency in reporting. For further information on Nature Portfolio policies, see our Editorial Policies and the Editorial Policy Checklist.

Statistics

For all statistical analyses, confirm that the following items are present in the figure legend, table legend, main text, or Methods section.

n/a	Confirmed	
☐	☒	The exact sample size (*n*) for each experimental group/condition, given as a discrete number and unit of measurement
☐	☒	A statement on whether measurements were taken from distinct samples or whether the same sample was measured repeatedly
☐	☒	The statistical test(s) used AND whether they are one- or two-sided<br>*Only common tests should be described solely by name; describe more complex techniques in the Methods section.*
☐	☒	A description of all covariates tested
☐	☒	A description of any assumptions or corrections, such as tests of normality and adjustment for multiple comparisons
☐	☒	A full description of the statistical parameters including central tendency (e.g. means) or other basic estimates (e.g. regression coefficient) AND variation (e.g. standard deviation) or associated estimates of uncertainty (e.g. confidence intervals)
☐	☒	For null hypothesis testing, the test statistic (e.g. *F*, *t*, *r*) with confidence intervals, effect sizes, degrees of freedom and *P* value noted<br>*Give P values as exact values whenever suitable.*
☒	☐	For Bayesian analysis, information on the choice of priors and Markov chain Monte Carlo settings
☒	☐	For hierarchical and complex designs, identification of the appropriate level for tests and full reporting of outcomes
☐	☒	Estimates of effect sizes (e.g. Cohen's *d*, Pearson's *r*), indicating how they were calculated

Our web collection on statistics for biologists contains articles on many of the points above.

Software and code

Policy information about availability of computer code

Data collection	No computer code was necessary for data collection.
Data analysis	All statistical analyses were conducted in R versions 4.2.3, 4.3.1, and 4.3.3, IBM SPSS Statistics for Windows, version 29.0 (IBM Corp.), or GraphPad Prism 9 software (GraphPad Software, Inc.). R Packages used were ChAMP, wateRmelon, ComBat, IlluminaHumanMethylationEPICanno.ilm10b4.hg19, Limma, Planet, DMRcate, missMethyl, REMP, DESeq2, ComBat-seq, and clusterProfiler.

For manuscripts utilizing custom algorithms or software that are central to the research but not yet described in published literature, software must be made available to editors and reviewers. We strongly encourage code deposition in a community repository (e.g. GitHub). See the Nature Portfolio guidelines for submitting code & software for further information.

Data

Policy information about availability of data

All manuscripts must include a data availability statement. This statement should provide the following information, where applicable:

- Accession codes, unique identifiers, or web links for publicly available datasets
- A description of any restrictions on data availability
- For clinical datasets or third party data, please ensure that the statement adheres to our policy

The datasets supporting the conclusions of the current study are included within the article and its additional files. Due to the sensitive nature of the patient data used, the data sets are not and cannot be made publicly available.

Human research participants

Policy information about studies involving human research participants and Sex and Gender in Research.

Reporting on sex and gender	Only the term "sex" has been used throughout the manuscript and the results of the sex-specific analyses have been reported. The information on sex is in the Supplementary material.
Population characteristics	The epiART cohort includes 80 newborns fertilized using ART (IVF n = 50 and ICSI n = 30) of which 42 using FRESH and 38 using FET embryos. Moreover, the cohort includes newborns from pregnancies of couples who had went through IUI (n = 12) as well as from SF (n = 11) couples who were about to start or already started ART treatment but got pregnant spontaneously. The naturally conceived controls (n = 77) were full term neonates born from uncomplicated pregnancies of healthy Finnish, Caucasian mothers.
Recruitment	Finnish, Caucasian couples applied to fertilization treatment in the Fertility clinic of the Family Federation of Finland or the Reproductive Medicine Unit of Helsinki University Central Hospital, Finland were recruited to this study and the samples were collected during the years 2015–2021. Control samples were collected during the years 2013–2015 in Helsinki University Hospital, Finland.
Ethics oversight	Ethics Committee of Helsinki University Central Hospital

Note that full information on the approval of the study protocol must also be provided in the manuscript.

Field-specific reporting

Please select the one below that is the best fit for your research. If you are not sure, read the appropriate sections before making your selection.

☒ Life sciences ☐ Behavioural & social sciences ☐ Ecological, evolutionary & environmental sciences

For a reference copy of the document with all sections, see nature.com/documents/nr-reporting-summary-flat.pdf

Life sciences study design

All studies must disclose on these points even when the disclosure is negative.

Sample size	This study is explorative in nature and all current samples in the cohort were used for the analyses. No statistical methods were used to predetermine sample size.
Data exclusions	No data were excluded from the analyses.
Replication	No replications on independent samples were done for the study.
Randomization	The study design did not require randomization.
Blinding	The study design did not require blinding.

Reporting for specific materials, systems and methods

We require information from authors about some types of materials, experimental systems and methods used in many studies. Here, indicate whether each material, system or method listed is relevant to your study. If you are not sure if a list item applies to your research, read the appropriate section before selecting a response.

Materials & experimental systems

n/a	Involved in the study	
☒	☐	Antibodies
☒	☐	Eukaryotic cell lines
☒	☐	Palaeontology and archaeology
☒	☐	Animals and other organisms
☒	☐	Clinical data
☒	☐	Dual use research of concern

Methods

n/a	Involved in the study	
☒	☐	ChIP-seq
☒	☐	Flow cytometry
☒	☐	MRI-based neuroimaging
